# A Predictive Model for Medical Events Based on Contextual Embedding of Temporal Sequences

**DOI:** 10.2196/medinform.5977

**Published:** 2016-11-25

**Authors:** Wael Farhan, Zhimu Wang, Yingxiang Huang, Shuang Wang, Fei Wang, Xiaoqian Jiang

**Affiliations:** ^1^ Health Sciences Department of Biomedical Informatics University of California - San Diego La Jolla, CA United States; ^2^ Department of Economics Boston University Boston, MA United States; ^3^ Department of Computer Science and Engineering University of Connecticut Storrs, CT United States

**Keywords:** clinical decision support, early classification, temporal phenotyping, clinical event context embedding

## Abstract

**Background:**

Medical concepts are inherently ambiguous and error-prone due to human fallibility, which makes it hard for them to be fully used by classical machine learning methods (eg, for tasks like early stage disease prediction).

**Objective:**

Our work was to create a new machine-friendly representation that resembles the semantics of medical concepts. We then developed a sequential predictive model for medical events based on this new representation.

**Methods:**

We developed novel contextual embedding techniques to combine different medical events (eg, diagnoses, prescriptions, and labs tests). Each medical event is converted into a numerical vector that resembles its “semantics,” via which the similarity between medical events can be easily measured. We developed simple and effective predictive models based on these vectors to predict novel diagnoses.

**Results:**

We evaluated our sequential prediction model (and standard learning methods) in estimating the risk of potential diseases based on our contextual embedding representation. Our model achieved an area under the receiver operating characteristic (ROC) curve (AUC) of 0.79 on chronic systolic heart failure and an average AUC of 0.67 (over the 80 most common diagnoses) using the Medical Information Mart for Intensive Care III (MIMIC-III) dataset.

**Conclusions:**

We propose a general early prognosis predictor for 80 different diagnoses. Our method computes numeric representation for each medical event to uncover the potential meaning of those events. Our results demonstrate the efficiency of the proposed method, which will benefit patients and physicians by offering more accurate diagnosis.

## Introduction

### Background

The large collection of healthcare data has brought tremendous opportunities and challenges to health care research [1]. The goal is to prevent and treat diseases by taking into account individual variabilities, which include genome, environment, and lifestyle [2]. There are many difficulties in making use of a large amount of health care data from heterogeneous sources with different characteristics (high dimensional, temporal, sparse, irregular, etc). The traditional data analysis methods (often developed for clean and well-structured data) do not fit these challenges well and may not be able to effectively explore the rich information in the massive health care data. Most of the existing models treat different medical events as distinct symbols without considering their correlations, and therefore are limited in terms of representation power [3-7]. For example, it is hard for those methods to use the correlation among different types of events (eg, the similarity between a prescription and a diagnosis, or an abnormal lab and a diagnosis). Indeed, many models assume a vector-based representation for every patient, where each dimension corresponds to a specific medical event. Such representation loses the temporal context information for each medical event, which could be informative for impending disease conditions.

Diagnoses share common symptoms making them enigmatic and hard to differentiate. Physicians might have a hard time discovering potential risks. Recent studies show that most diagnostic errors have been associated with flaws in clinical reasoning and empirically prove the evidence between cognitive factors and diagnostic mistakes [8,9]. In 25% of the records of patients with a high-risk diagnosis, high-information clinical findings were present before the high-risk diagnosis was established [10]. Our predictive model aims to counterbalance cognitive biases by suggesting possible diagnoses based on the patient's medical history. We combine data from different sources in an innovative way, which synthesize information more comprehensively than existing models. Our model is more accurate than most predictive models in the literature and it is less computationally expensive.

With the above considerations, we introduced a new representation for electronic health records (EHR) that was context-aware and combines heterogeneous medical events in a uniform space. Here, the “context” was defined with respect to each medical event in the patient EHR. The context around an event *A* is the order of medical events happening before and after *A* within the patient EHR corpus. For each patient, through the concatenation of all medical events in his or her EHR according to their sequential timestamps (without considering the order of tied events), we obtained a “timeline” describing all historical conditions of the patient. While generating context, we lost the exact time at which each event occurred. Therefore, the context around a specific medical event in the timeline was similar to the context around a word in a narrative text.

How to derive effective word representations by incorporating contextual information is a fundamental problem in natural language processing and has been extensively studied [11-13]. One recent advance is the “Word2Vec” technique that trains a 2-layer neural network from a text corpus to map each word into a vector space encoding the word’s contextual correlations [14,15]. The similarities (usually computed by the cosine distance over the embedded vector space) reflect the contextual associations (eg, words *A* and *B* with high similarity suggest that they tend to appear in the same context). Word2Vec is able to extract event semantics despite the relatively small training corpus. We extended Word2Vec to support dynamic windows to handle the temporal nature of medical events.

Based on the contextual embedding representation, we developed 3 models to predict the 80 most common diagnoses based on Medical Information Mart for Intensive Care III (MIMIC-III) dataset. The goal of this study was to predict the onset risk of each diagnosis based on historical patient records. Our model achieves an area under the receiver operating curve (ROC) curve (AUC) higher than 0.65 for half of the 80 diagnoses. We further introduced time decay factors in the model to reflect the fact that more recent events have a bigger impact on the prediction. Our model was also able to learn bioequivalent drugs (and medical events) and build the semantic relationship, which cannot be fulfilled with most existing predictive models.

In this paper, we encountered a more challenging task than previous work mentioned in the next section. Here, we built a novel diagnosis predictor, which means our model was predicting diagnoses that do not occur in patient history. Most of chronic disease will eventually be listed on every admission for that patient, predicting the same diagnosis again will enhance the performance of our predictor but will not add anything new for the physician treating that patient. Nonetheless, we ran predictor against all diagnoses (ie, not restricted to novel ones) to be able to compare it with previous work. We achieved a mean AUC of 0.76 for 80 diagnoses.

### Previous Work

A substantial amount of work has been conducted on systems to support clinical decisions using predictive models. For example, Gottlieb et al [3] proposed a method for inferring medical diagnoses from patient similarities using patient history, blood tests, electrocardiography, age, and gender information. However, their method can only predict discharge codes at international classification of diseases (ICD)-9 level 1, which are relatively generic and cannot differentiate among a wide range of diverse diagnoses. In risk prediction with EHR, Cheng et al [16] used convolutional neural network with a temporal fusion to predict congestive heart failure and chronic obstructive pulmonary disease within the next 180 days. Their approach can only handle 2 diagnoses and achieved an AUC of less than 0.77. Ghalwash et al [17] extracted multivariate interpretable patterns for early diagnosis. They constructed key shapeletes (a time series subsequence) to represent each class of early classification using an optimization-based approach. This technique is computationally expensive and would not work efficiently with a large dataset, therefore, they only focused on a small number of diagnoses. By taking advantage of a different set of inputs, functional magnetic resonance imaging (fMRI) images, Wang et al proposed high-order sparse logistic regression and multilinear sparse logistic regression [18,19] for early detection of Alzheimer disease and congestive heart failure. Their results surpassed standard learning algorithms, such as nearest neighbor, support vector machines (SVM), logistic regression (LR), and sparse logistic regression. But not all patients have fMRI images within EHR, thus their models are only limited to a small subset of patients. Taslimitehrani et al [20] constructed a logistic regression model using CPXR(log) method (short for contrast pattern aided logistic regression) to predict mortality rate in heart failure patient. They consulted a cardiologist and a cardiovascular epidemiologist to identify patient cohort from EHR data collected from patients admitted to the Mayo Clinic between 1993 and 2013. Their model is specific and can only be extended to different diagnoses after consulting specialists. Recently, Lipton et al [21] used long short-term memory (LSTM) recurrent neural network for a multilabel classification of diagnosis in the pediatric intensive care unit, which demonstrated improved performance over a set of standard learning methods. They trained LSTM neural network (ie, a special recurrent neural network, which has a forget gate to capture long-term dependency) on variable length inputs of large size. Nevertheless, their model is a black box, which cannot be interpreted by human experts.

There is also some related work on feature representation. Tran et al [22] presented a generative model based on nonnegative Restricted Boltzmann Machine to learn low-dimensional representations of the medical events from electronic medical records (EMRs). Their model assumes EMRs are aggregated into regular time intervals and captures the global temporal dependency structures of the events. Another work by Che et al [23] explored deep learning applications to the problem of discovery and detection of characteristic patterns of physiology in clinical time series. They applied deep feed-forward neural network with fully connected layers using graph Laplacian priors and developed an efficient incremental training procedure to detect physiological patterns of increasing length, which demonstrated good AUCs. Using a similar approach, Liu et al [24] extracted temporal phenotypes from longitudinal EHR using a graph-based framework. They represented each patient’s history using a temporal graph, where each node serves as a medical event and edges are constructed based on the temporal order of events. Using those temporal graphs, they identified the most significant and interpretable subgraph basis as phenotypes, which is used later as a feature set for their predictive model. But their method has only been applied to a small cohort associated with congestive heart failure.

The context-aware representation proposed in this paper provides a new way of combining data and building predictive models. We developed several methods on top of the novel representation and achieved a high AUC. As mentioned earlier, none of the previous work tackled the challenge of predicting a novel diagnosis. In this paper, we show that our model is able to predict a diagnosis that was not previously identified. Also, our model is highly generalizable, which can predict multiple diseases without having to tune parameters for each one of them.

## Methods

### Temporal Sequence Construction

In this section, we will present the proposed sequential prediction framework by starting with explanation about what the components of a sequence are and how the sequential prediction is formulated.

**Figure 1:**
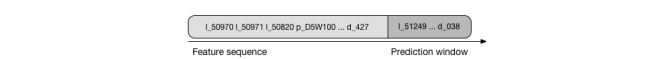
Sequence construction.

In our model, a sequence was defined as a combination of lab tests, prescriptions, and diagnoses that were performed, ordered, or assigned to a patient in multiple hospital admissions. Lab tests and prescriptions were represented by unique identifiers defined by the dataset. But because two tied events could have the same identifier we added ‘ *l* _’ at the beginning of lab tests key and ‘ *p* _’ for prescriptions. Diagnoses, on the other hand, were all represented with their ICD-9 code prefixed with ‘ *d* _’. To conserve part of the temporal information, we sorted those events from oldest to latest. Hence, we lost the exact timestamp at which the event happened. A patient sequence contained data from multiple admissions that happened within a year apart from each other. We sliced the most recent admission out of the sequence and used its diagnoses as gold standard in the prediction phase, while preceding admission events are used as features. A graphical illustration of a sequence is depicted in Figure 1.

Unlike earlier work, in this paper we did not preprocess diagnosis ICD-9 level to generalize them at one level. Instead, we kept the ICD exactly as identified by the physician. For example, “pneumonia” (486) is a level 3 diagnosis and “anemia in chronic kidney disease” (285.21) is a level 5; all were kept as unique events in the same sequences. This way, our predictor will identify the diagnosis in the same specificity level as diagnosed by the physicians.

Also, due to the nature of medicine, some medical events are extremely rare in the population. Hence, it would be hard to extract common patterns from a very small sample. For our experiments, we excluded events that appear in less than 1% of the total number of sequences.

### Contextual Embedding

Word2Vec [15], a tool created to learn word embeddings from a large corpus of text, has recently gained popularity. It has mainly been applied in natural language processing to generate continuous vector representation for each word. The distances between these words (in the vector space) describe the similarities of those words. A well-known example of the so-called “semantic relationship” presented in the original paper is that queen to king has almost same distance like woman to man [15]. Another popular semantic relationship learned using the same model is reported as “V[France] – V[Paris] ≈ V[Germany] – V[Berlin]” [8], where *V* is the vector representation of the word.

**Figure 2:**
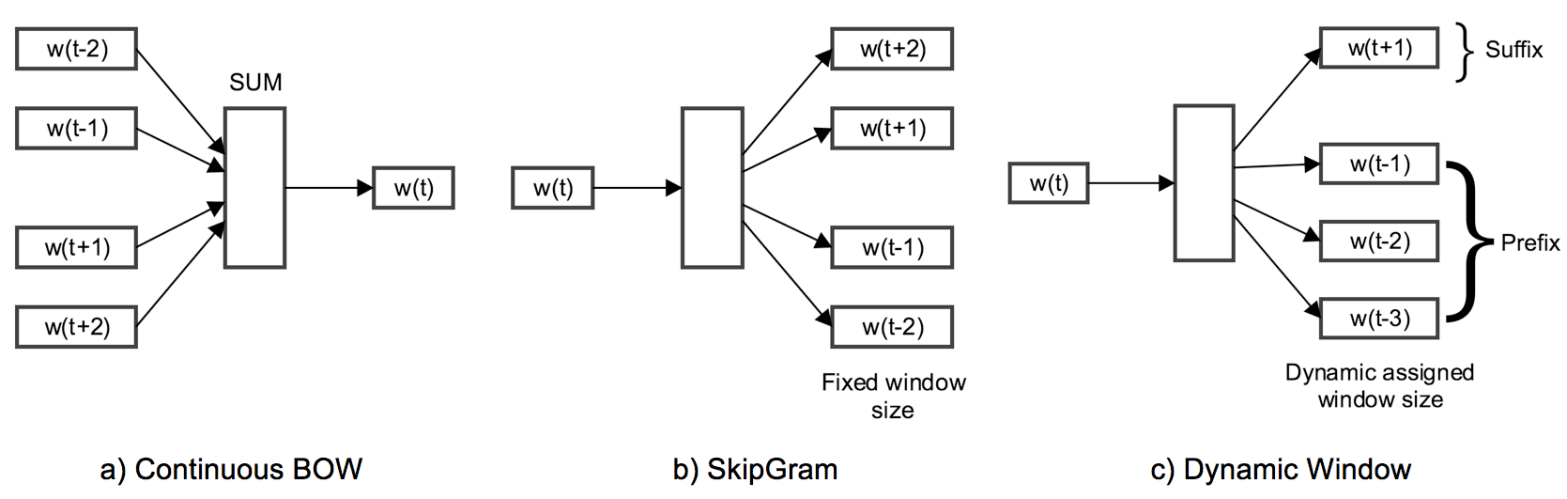
Different Word2Vec modes. (a) and (b) are the Continuous Bag of Words (CBOW) and SkipGram modes, which have been widely used in neurolinguistic programing (NLP) problems; (c) a new and more flexible mode to support models using dynamic window.

Word2Vec, in its core, depends on 2 parameters: size and window; size defines the dimensionality of the vector representation, while window is the maximum distance between a word and its predicate word in one sentence. Word2Vec supports 2 modes of operation [25]: (1) Continuous Bag of Words: the input to the model is a collection of words, and the model would predict the missing word, and therefore, it can predict a word given its context as illustrated in Figure 2 a; and (2) Skip-Gram: the target word is now in the input to the model, and the context words are going to be predicted, as illustrated in Figure 2 b.

In the proposed model, we extend Word2Vec to support one extra mode as follows: Dynamic Window: a customized mode in our experiment defines different windows for words in the sequence as prefix (preceding words) and suffix (succeeding words) as illustrated in Figure 2 c.

In our paper, we used Word2Vec to generate vector representation for each medical event by feeding it with the medical event sequences discussed in the previous section. With Word2Vec technique, we can extract event semantics from a relatively small corpus.

### Learning Methods

We present the proposed predictive methods in this section. For each method, we used the training set to learn binary classification models for diagnoses of interest. Those binary classification models calculate the probability of having a future diagnosis given test sequences. A test sequence will end up with multiple predictions, one for each diagnosis. Each diagnosis prediction is completely independent from other diagnoses, formulating our approach as multiclass classification problem. All learning methods make use of the contextual representation generated by Word2Vec. We passed patient sequences from the training set into Word2Vec to learn a contextual vector representation for each medical event.

#### Collaborative Filtering

In this method, we leveraged a recommendation system [26] that calculates patient-patient projection similarity. Each patient record in a training set was projected into the vector space by summing up event vectors in its sequence multiplied by the temporal factor. Intuitively, patients with similar history projections are more likely to foretell the future more than others. This information was used in the decision of what diagnosis a patient might get.

**Figure 3:**
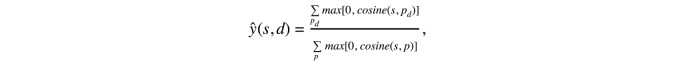
Collaborative filtering weighted voting.

For prediction, we projected the test patient sequence exactly like training records. Then, we found the patients with the most similar projections. We calculated the probability based on weighted voting, where the weight is the cosine similarity of the 2 patients (Figure 3).

Where *s* is a patient sequence, *d* is a diagnosis, *p*_d_ corresponds to all patients in training set who end up with diagnosis *d*, and *p* corresponds to all patients.

#### Patient-Diagnosis Event Similarity

In the patient-diagnosis event similarity (PDES) prediction method, we used the generated vector representation to build *S*, a cosine similarity matrix. *S* is a (*N×D*) matrix, where *N* is the number of all medical events and *D* is the number of diagnoses. For example, *S['d_428','l_50862']* is the cosine similarity between heart failure and albumin blood test.

**Figure 4:**
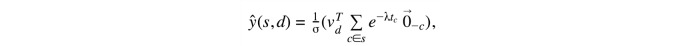
Patient-diagnosis event similarity.

To predict the diagnosis given in a patient sequence, we first generated patient event vector of length N by simply summing one-hot representation (eg, mapping the medical events to vectors of length N, where the n^th^ digit is an indicator of that medical event) of its events multiplied by temporal factor, to emphasize recent events. Then, we use this array to find the similarity of that patient with a particular diagnosis using the equation in Figure 4.

**Figure 5:**
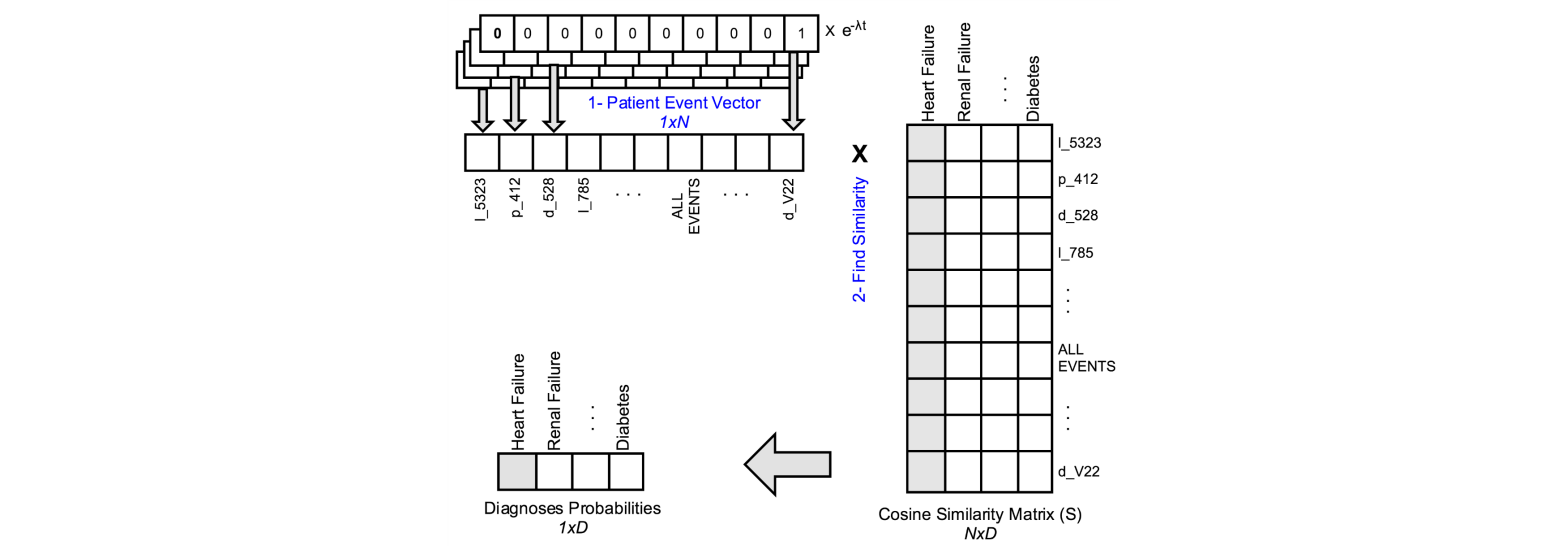
Patient diagnosis event similarity.

Where *s* is a patient sequence, *d* is a diagnosis, σ is a normalization constant, *v*_d_ is a column in the similarity matrix corresponding to the diagnosis *d*, *c* is a medical event, *λ* is the decay factor and *t*_c_ is time passed from the latest event. is the one-hot vector representation of *c*. The term *e*^-λtc^ is used to account for the decay of impact of medical histories like in the previous example. Figure 5 depicts the prediction methodology of PDES.

The higher the similarity, the more likely a patient will get the diagnosis in the next visit. It is possible to get negative similarity values, but empirical evaluation showed that converting negative similarities to zero achieved better performance. There are very few hyperparameters that need tuning: Word2Vec size and window parameters, and *λ*, the decay factor.

**Figure 6:**
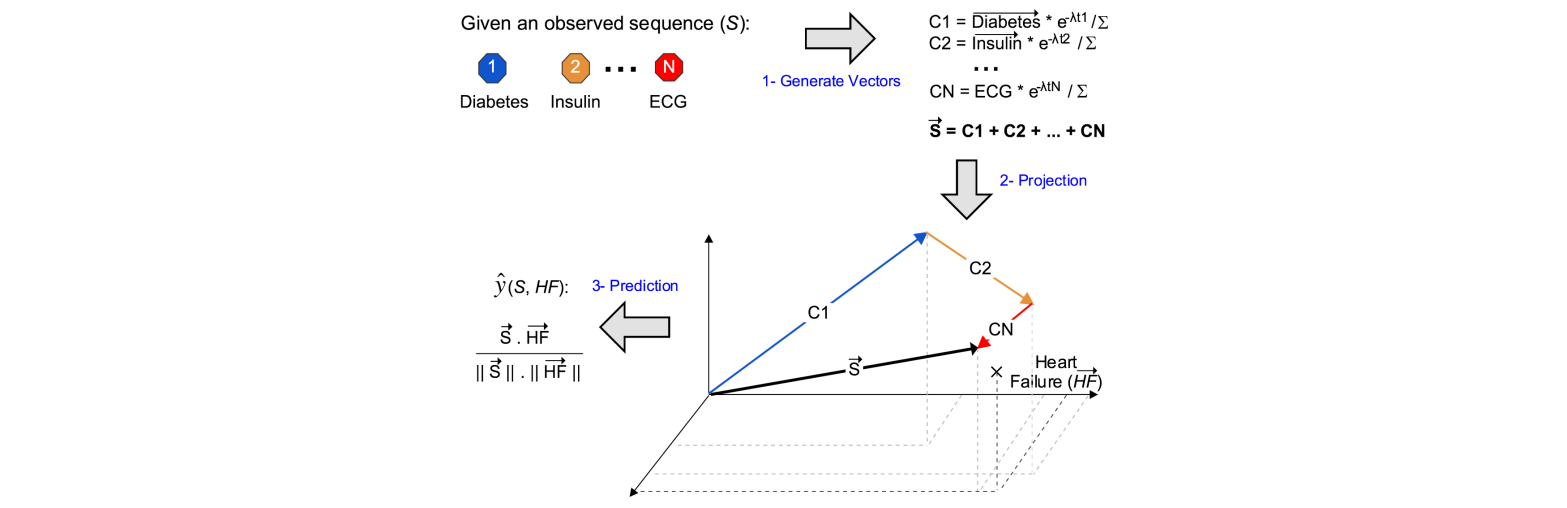
Patient-diagnosis projection similarity, where Σ is the summation of temporal factors.

One of the issues with this approach is that it does not take full advantage of the semantic similarity. Consider 2 similar prescriptions, the cosine similarity of them with respect to a particular diagnosis will be almost the same. If a patient happens to be treated with the first prescription and not the second, then, the patient representation will have a value of zero at the one-hot representation of the second prescription. Hence, the result of the dot product in Figure 6 will falsely diminish, reducing the probability of that diagnosis. We will overcome this problem in the next method.

#### Patient-Diagnosis Projection Similarity

**Figure 7:**
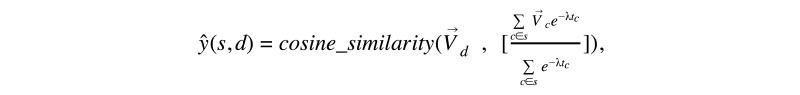
Patient-diagnosis projection similarity.

Based on the vector representation, we also proposed another prediction method, patient-diagnosis projection similarity (PDPS), where we project patient sequence into the vector space, bearing in mind the temporal impact. Then, we computed the cosine similarity between the patient vector and the diagnosis vector. The equation in Figure 7 demonstrates the prediction method.

Where the is the vector contextual representation of diagnosis *d* in the vector space, and is the vector contextual representation of a medical event in patient sequence. Figure 6 illustrates the prediction methodology used in PDPS similarity method. PDPS can solve the problem of nonidentical similar events faced by PDES. Here, patient projection is unaffected by similar events; whether the patient got the first prescription or the second, PDPS would still add an equivalent vector into the patient projection.

## Results

### Data (Medical Information Mart for Intensive Care III)

In this section, we evaluate the proposed methods with a real-world dataset. We present the results of these experiments and discuss the choice of hyperparameters. We also compare the results of different models, diagnoses, and datasets. We compare our results with standard learning methods/algorithms that do not make use of the contextual representation. We will begin this section by introducing the dataset we used.

To test the proposed methods, we explored MIMIC-III database [27], which contains health-related data associated with 46,520 patients and 58,976 admissions to the intensive care unit of Beth Israel Deaconess Medical Center between 2001 and 2012. The database includes detailed information about patients, such as demographics, admissions, lab test results, prescription records, procedures, and discharge ICD-9 diagnoses.

Because we wanted to predict the next diagnosis, we excluded the patients who were only admitted once. We also eliminated rare lab tests and prescriptions that only happened in less than 50 admissions. In total, we select 204 most common lab events that flagged as abnormal, 1338 most common prescriptions, 826 most common diagnoses, 274 most common conditions, and 171 most common symptoms. After applying the method introduced in the Temporal Sequence Construction section, we constructed 5642 temporal sequences using medical records of 5195 patients. The total number of sequences was larger than the number of patients because we used a threshold of 1 year as the medical history cut-off. Hence, a patient could have multiple sequences if admissions happen more than 1 year apart.

### Baselines

As a sanity check, we needed a proof to make sure that our models were more beneficial than common learning models. So we decided to compare our results with baseline models. First, we converted medical events into one-hot representation vectors. Then, we generated patient vectors by summing up the one-hot representation vectors of its events. These vectors served as input features, while the label was the binary value that indicated whether a diagnosis was found in the last admission. We generated one label vector for each diagnosis and ran our learning algorithm once for each diagnosis.

We explored multiple baseline models by passing our features through SVM, LR, and decision trees learning models. We also applied decay factor just as described in PDES model. LR with decay was able to achieve the highest results. Hence, we decided to adopt it as our baseline.

### Performance

We applied the 4 methods to the MIMIC dataset. We adopted AUC, accuracy, and *F*-score as measurements to compare different models. We used 10-fold cross-validation to evaluate each model.

**Figure 8:**
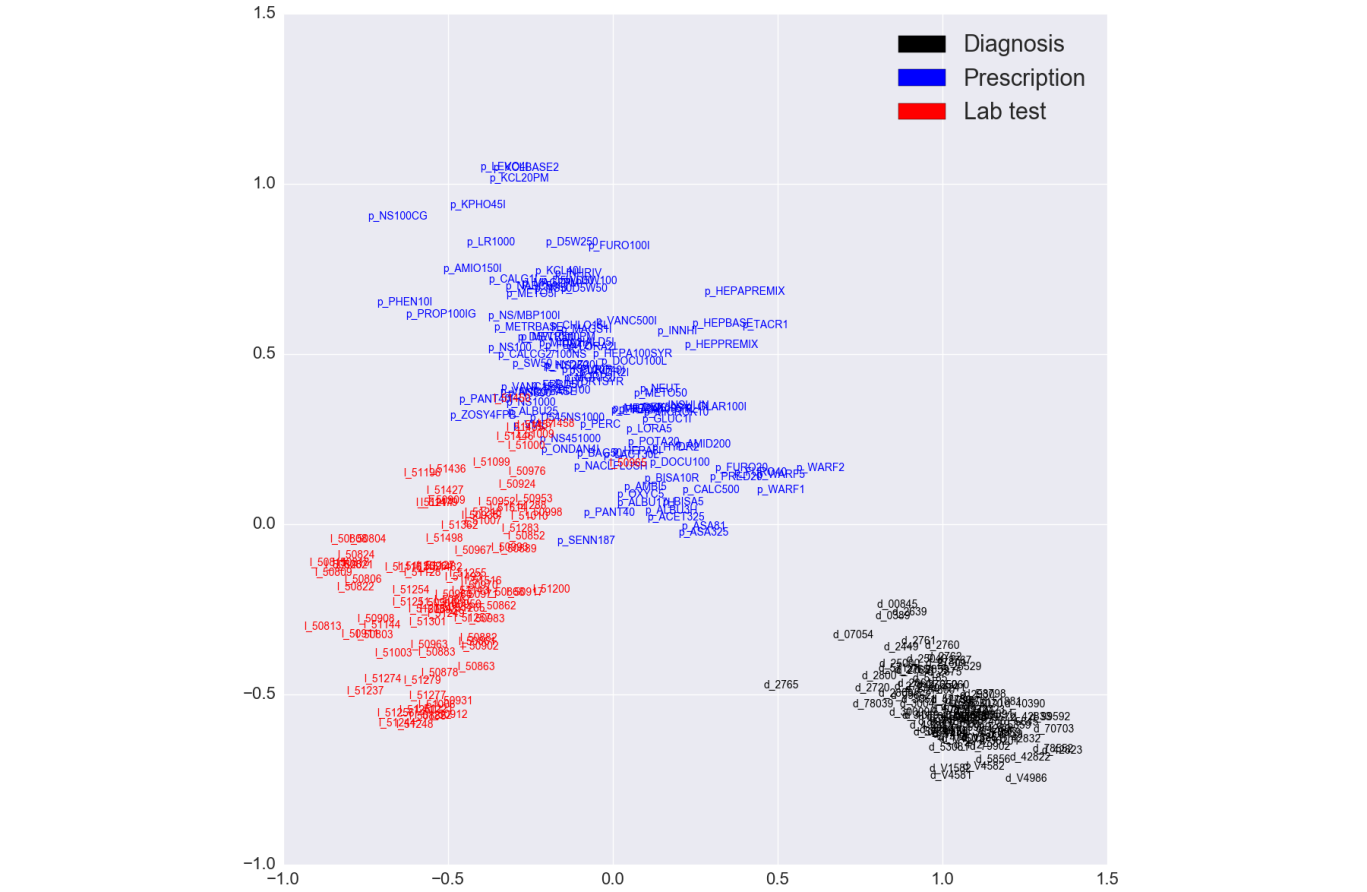
Medical event contextual representation displayed in 2 dimensions for visualization purposes. These dimensions are arbitrary learned by Word2Vec.

Other than the baseline model LR, all models we proposed incorporated the vector representations of medical events from Word2Vec. For visualization purposes, we limited the dimensions of the hyperspace to 2 dimensions. Figure 8 illustrates the limited contextual representation color coded by event type. Vector representations constructed by Word2Vec were able to capture semantic meaning of medical events. Word2Vec clusters events based on their type as shown in the figure. In addition, it was able to capture closely similar events, for example, the cosine similarity of *’p_WARF2’* (Warfarin 2-mg Tab) and *’p_WARF1’* (Warfarin 1-mg tab) was 0.924. All prescriptions starting with *’p_WARF’* were close to each other around the point (0.5, 0.2). This representation simplifies learning because it groups similar events by unified content.

**Figure 9:**
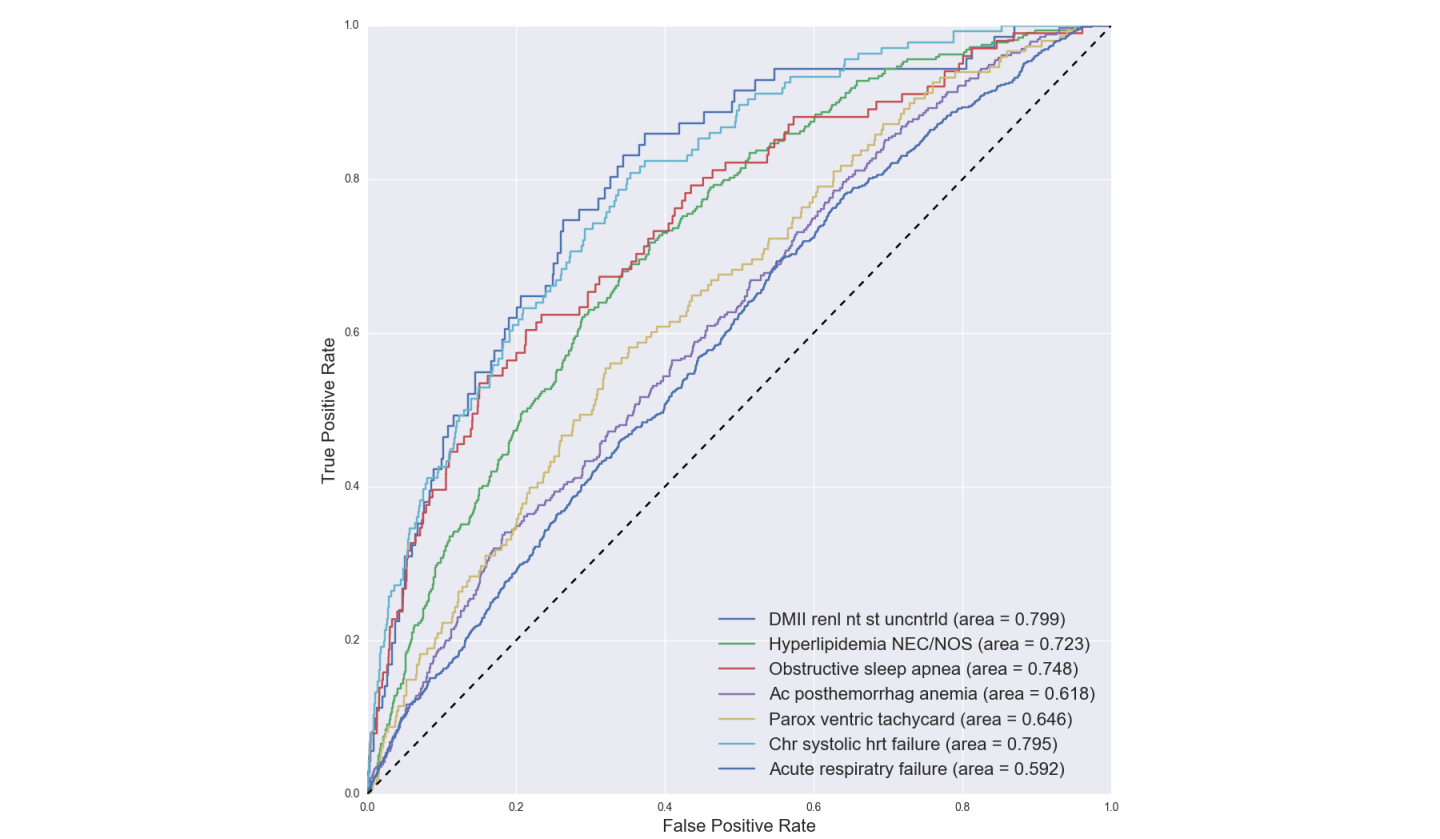
Patient–diagnosis projection similarity (PDPS) receiver operating characteristic (ROC) curves and their corresponding area under the receiver operating characteristic curve (AUCs) for each disease prediction.

Our experiments included predicting the 80 most common diagnoses for each patient. More formally, we constructed a multilabel classification problem where each patient sequence could be labeled with multiple diagnoses. A patient is labeled with a diagnosis if and only if that particular diagnosis happens in the final admission (ie, prediction window). We selected 4 diagnoses to discuss in the paper, which are displayed in Table 1 with AUC for each diagnosis in each model. From the table, it is noticed that PDPS achieves the highest performance in most cases. The full results can be found in Multimedia Appendix 1. Figure 9 contains 7 selected ROC curves collected from the entire 80 diagnoses. This figure shows how our learning method performs differently on various diagnoses.

**Table 1:** Sample results of MIMIC-III.

	Chronic systolic heart failure (485.22)			Acute posthemorrhagic anemia (285.1)			Hyperlipidemia not elsewhere classifiable/not otherwise specified (272.4)			Septicemia not otherwise specified (038.9)		
	AUC^a^	ACC^b^	*F*-score	AUC	ACC	*F*-score	AUC	ACC	*F*-score	AUC	ACC	*F*-score
LR^c^	0.780	0.947	0.237^d^	0.550	0.779	0.159	0.699	0.737	0.224	0.593	0.651	0.217
CF^e^	0.784	0.849	0.145	0.581	0.815^d^	0.152	0.733^d^	0.772	0.254^d^	0.641	0.632	0.225
PDES^f^	0.793	0.869	0.158	0.579	0.408	0.153	0.702	0.763	0.221	0.648	0.682	0.239
PDPS^g^	0.795^d^	0.953^d^	0.213	0.618^d^	0.786	0.175^d^	0.723	0.851^d^	0.238	0.652^d^	0.720^d^	0.242^d^

^a^AUC: area under the receiver operating characteristic curve.

^b^ACC: accuracy.

^c^LR: logistical regression.

^d^The highest value between the four different methods.

^e^CF: collaborative filtering.

^f^PDES: patient-diagnosis event similarity.

^g^PDPS: patient-diagnosis projection similarity.

**Figure 10:**
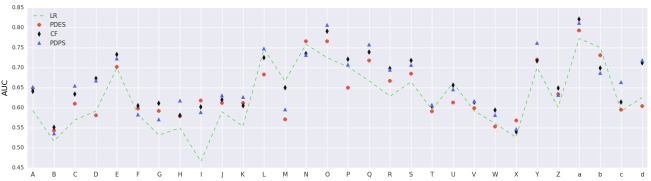
Patient-diagnosis projection similarity (PDPS) area under the receiver operating characteristic curve (AUC) of 30 diagnoses on the medical information mart for intensive care III (MIMIC III) dataset. (A) septicemia NOS, (B) hypothyroidism not otherwise specified (NOS), (C) protein-cal malnutr NOS, (D) pure hypercholesterolem, (E) hyperlipidemia not elsewhere classifiable (NEC)/NOS, (F) hyposmolality, (G) acidosis, (H) Ac posthemorrhag anemia, (I) anemia-other chronic dis, (J) thrombocytopenia NOS, (K) depressive disorder NEC, (L) obstructive sleep apnea, (M) hypertension NOS, (N) Hy kid NOS w cr kid I-IV, (O) Hyp kid NOS w cr kid V, (P) old myocardial infarct, (Q) Crnry athrscl natve vssl, (R) atrial fibrillation, (S) congestive heart failure NOS, (T) pneumonia, organism NOS, (U) Chr airway obstruct NEC, (V) food/vomit pneumonitis, (W) pleural effusion NOS, (X) pulmonary collapse, (Y) cirrhosis of liver NOS, (Z) acute kidney failure NOS, (a) end-stage renal disease, (b) chronic kidney dis NOS, (c) osteoporosis NOS, and (d) Surg compl-heart.

The outcome of each binary diagnosis predictor was a probability between 0 and 1. We computed a distinct threshold for each diagnosis, above which a patient was labeled as positive. The threshold was calculated such that it optimizes the F1 score (ie, Youden index [28]). Finally, the accuracy gets computed after labeling test patients. Figure 10 displays AUC results of 30 different diagnoses using PDPS. As can be seen from the graph, our results are robust across diagnoses and models, and demonstrated clear performance advantage over other methods in comparison.

We investigated how our predictor works by analyzing the true positive sequences of patients to find a medical justification behind each diagnosis. For each diagnosis, we computed the top medical events that our predictor used as the leading cause. Most findings were precise and clinically insightful (thanks to our medical doctor collaborators for examination). We list a few examples here. Chronic kidney disease (CKD) is predicted after finding late manifestations of joint, soft tissue, and bone problems coexist (musculoskeletal). In addition, over the counter pain killers (nonsteriodal ant-inflammatory drugs), can cause CKD; however, this problem often goes unrecognized by health care providers, especially when they do not check kidney function. Another example is pneumonia, where our predictor associates glossitis, which can lead to problems in protecting patients’ airways, with pneumonia. Chest deformity can damage blood vessels (capillaries) in the lungs, allowing more fluid to pass into the lungs, making the patient more sensitive to bacteria, viruses, fungi, or parasites infections. Vocal cord diseases can also lead to pneumonia so as autosomal anomalies where abnormal chromosomes make patients at increased susceptibility to respiratory disease like pneumonia and other infectious disease. Another example, obstructive sleep apnea is predicted through structural and mechanical problems like acute tracheitis without mention of obstruction, scoliosis, and obesity, in addition to inflammation in the nasal membranes like allergic rhinitis and poisoning by opiates and related narcotics, which cause sleep disturbance and hypoventilation (decrease in respiratory rate).

Yet another example is that cirrhosis of the liver without ETOH linked with several hepatitis C disorders. Hepatitis C can be a precursor to nonalcoholic cirrhosis. Malignancy of the rectosigmoid junction would rarely cause cirrhosis, but can sometimes result in liver metastasis that can cause laboratory abnormalities similar to those found in cirrhosis–that is why our predictor slightly linked them together. Our predictor was able to learn patterns of these diagnoses without the supervision of a medical practitioner.

#### Decay - Temporal Effect

**Figure 11:**
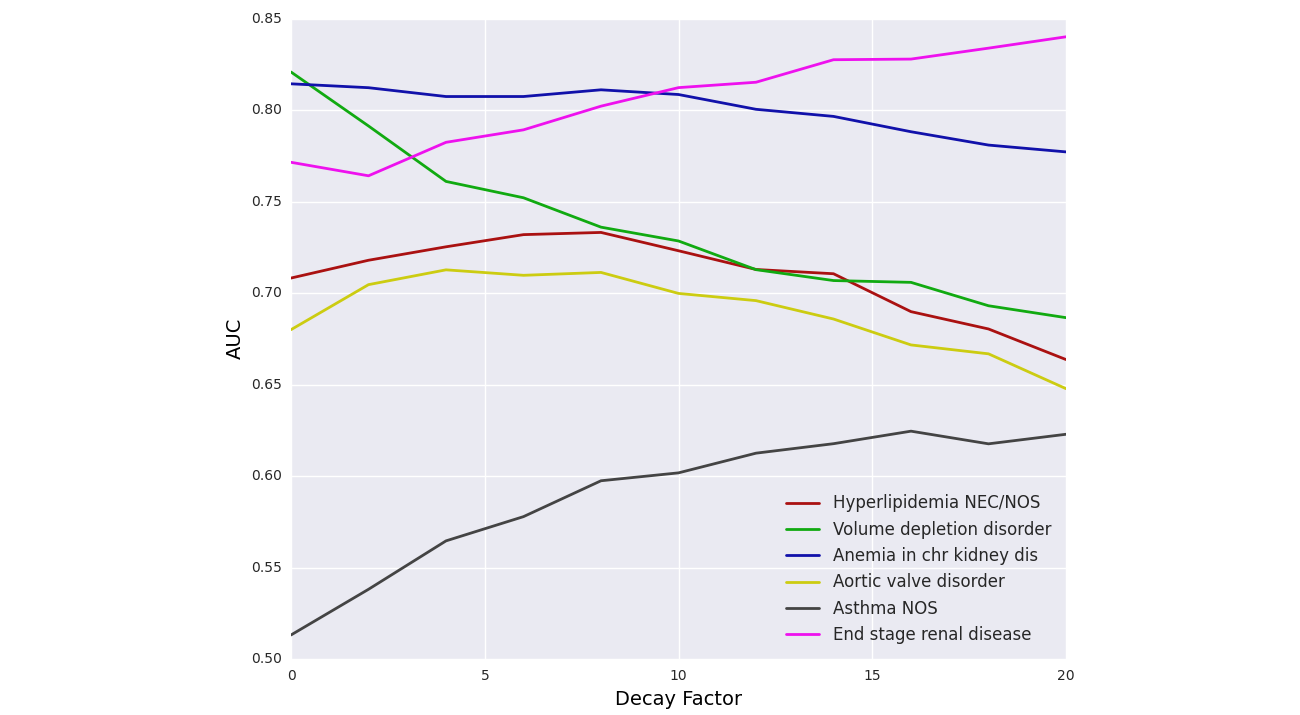
Effect of decay on patient-diagnosis projection similarity (PDPS) similarity.

Figure 11 illustrates the effect of adding temporal factor to the PDPS prediction model. Adding temporal factor forced the model to focus more on the recent events and to leave older ones with less influence. The main observation here is that different diseases behave distinctly. Some diagnoses like “volume depletion disorder” and “anemia” decreased in AUC as we increased decay factor, which means that those diseases are predicted more accurately by looking at the entire patient history. Others like “end-stage renal disease” increased AUC when increasing the decay factor, which implies that the model had to focus on the last few events to be able to predict it. Most of the diagnoses like “aortic valve disorder” and “hyperlipidemia” had a bell shaped curve with different optimal decay value. This phenomenon applies to all methods including the baseline.

#### Data Balancing

Health data are often uneven where some diagnoses are more common than others. For example, in the MIMIC-III dataset, “gout” is less common than “congestive heart failure.” This can affect the downstream predictive models and we tried to mitigate it by balancing the dataset. However, balancing the dataset was not an easy task because admissions tended to be labeled with multiple diagnoses, for example, diabetes (ICD-9: 250.00) and congestive heart failure (ICD-9: 428.0). Therefore, when we try to balance an infrequent diagnosis, by duplicating some of its sequences randomly, we increase the rate of other diagnoses that happened with the infrequent one. We approximated the balance by making sure that each diagnosis appeared in at least 8% of the total sequences. This step was done by duplicating random samples that contained infrequent diagnoses until all diagnoses passed the 8% threshold.

**Figure 12:**
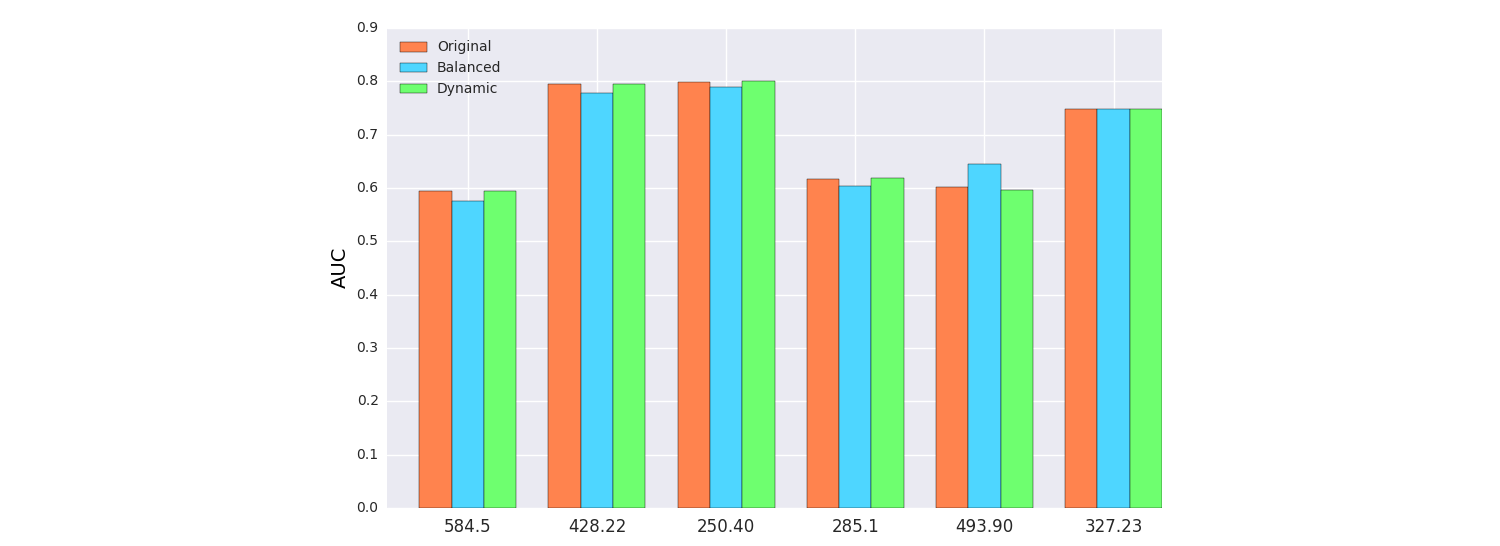
Effect of balancing the dataset on patient-diagnosis projection similarity (PDPS). 584.5 Ac kidny fail, tubr necr, 428.22 Chr systolic hrt failure, 250.40 DMII renl nt st uncntrld, 285.1 Ac posthemorrhag anemia, 493.90 Asthma NOS, 327.23 Obstructive sleep apnea.

As shown in Figure 12, balancing had small impact on the overall performance. Context representation did not change a lot from adding the same sequence again, and that explains why our model did not benefit from rectifying skewness.

#### Dynamic Window

Recall that dynamic window defines different window sizes for each word in the sequence. In PDPS, we defined the window to be 365 days, so any medical event that happened before that would be discarded, so that they have no influence on the contextual representation. As can be seen from Figure 12, there is a minor impact on overall performance because we believed that the dynamic window was being overshadowed by the temporal decay. In other words, the influence of old events was limited due to our adaptation of the temporal factor, eliminating it by dynamic window was not going to bring a significant change.

The results show that a predictive models using semantic extraction worked better than baseline learning methods. The PDPS method achieved the highest mean performance across 80 different diagnoses. Each diagnosis reached its highest AUC on a different decay constant lambda, this variation depended on the nature of the disease. We also exposed different variations that included dynamic window and balancing the dataset.

## Discussion

### Limitations and Future Work

The proposed studies have several limitations. When making predictions for our datasets, we neglected demographic information such as age, gender, and race. One way of incorporating this information is by injecting extra words in the sequences, for example, gender could be represented as *'g_Male'* and *'g_Female'*. We believe that some demographic information is already embedded within the medical event vector representation, for example, normal delivery (with ICD-9 code: 650) would also imply that the patient is a female. Therefore, adding vocabulary to explicitly identify the demographics may not improve the model significantly. We will test this hypothesis in future work.

Most learning models deal with a group of hyperparameters like decay factor, window, size, and space dimension. Tuning those parameters consumes a considerable amount of time and effort, especially for collaborative filtering. PDES and PDPS are substantially faster so we are able to tune the parameters and reuse them for collaborative filtering method.

Medical error is one of the issues with which all early prognosis predictors have to deal [26]. Medical error might include misdiagnosis, delayed, inaccurate, or incomplete diagnosis. Diseases related to inflammation, autoimmune, or mild infection (with ICD-9 codes: 424, 507, 511, etc) has no specific symptoms; need extensive lab work; and could still be incorrectly analyzed. When training contextual representation, a sequence in the training set with misdiagnosis could slightly modify vector projection of medical events, which might be negligible. On the other hand, a misdiagnosed test sequence could alter the overall performance. There are some diseases, such as pneumonia (486) and septicemia (038), which develop quickly and do not have a history pattern. Thus PDPS does not do very well (AUC slightly over 0.60 for those difficult cases). We might need to develop new and customized models to predict these special cases.

Another limitation of our approach is that it assumes the sequence events are sampled at the same frequency (without considering the order of tied events), which means the temporal effect is not accurately represented. We can solve this problem by incorporating each event with timestamp in combination with dynamic window for the accurate representation.

### Conclusion

We developed a sequential prediction model of clinical phenotypes based on contextual embeddings of medical events. Using the vector representation as features for our PDPS model, we were able to achieve a mean AUC of 0.67 and a median AUC of 0.65 (AUC ranging between 0.54 and 0.85) on 80 diagnoses from MIMIC dataset. The results demonstrated that learning EHR could benefit from abstracted contextual embeddings, which also preserved the semantics for human interpretation.

Our approach suggested a new way to learn EHR using contextual embedding methods, where we believe there is still much to discover. In this paper, we explored a set of prediction methods that exploit medical event embeddings. The experimental results showed that our best predictor is able to efficiently learn 14,080 medical cases with 10-fold cross validation under 15 minutes as well as achieved an AUC better than most state-of-the-art methods. We recognize that some diagnoses are still hard to predict either due to their medical complexity and wind up misdiagnosed or due to their sudden unexpected nature. In future work, we plan to focus on making temporal factors more accurate and fusing demographic information within patient medical event sequences.

## Appendix

Multimedia Appendix 1 MIMIC III Results for All 80 Diagnoses.

## Abbreviations

AUC: area under the receiver operating characteristic curve
CKD: chronic kidney disease
EHR: electronic health records
LSTM: long short-term memory
EMR: electronic medical records
fMRI: functional magnetic resonance imaging
LR: logistic regression
MIMIC-III: medical information mart for intensive care III
PDES: patient-diagnosis event similarity
PDPS: patient-diagnosis projection similarity
ROC: receiver operating characteristic
SVM: support vector machine
