# Supplementary material for: A Predictive Model for Medical Events Based on Contextual Embedding of Temporal Sequences
Source: JMIR Med Inform. 2016 Nov 25;4(4):e39. doi: 10.2196/medinform.5977 (PMC5148810; doi:10.2196/medinform.5977)
Supplement: Multimedia Appendix 1 [file medinform_v4i4e39_app1.pdf]

| ICD | Diagnosis | LR | Collaborative | PDES | PDPS |
|---|---|---|---|---|---|
| 008.45 | Intestinal infection due to Clostridium difficile | 0.5496 | 0.6098 | 0.6230 | 0.6264 |
| 038.9 | Unspecified septicemia | 0.5926 | 0.6406 | 0.6478 | 0.6515 |
| 070.54 | Chronic hepatitis C without mention of hepatic coma | 0.8083 | 0.7837 | 0.7160 | 0.8530 |
| 244.9 | Unspecified acquired hypothyroidism | 0.5167 | 0.5511 | 0.5426 | 0.5360 |
| 250.00 | Diabetes mellitus without mention of complication, type II or unspecified type, not stated as uncontrolled | 0.6629 | 0.6733 | 0.6490 | 0.6099 |
| 250.40 | Diabetes with renal manifestations, type II or unspecified type, not stated as uncontrolled | 0.7674 | 0.8213 | 0.7801 | 0.7989 |
| 250.60 | Diabetes with neurological manifestations, type II or unspecified type, not stated as uncontrolled | 0.7290 | 0.7945 | 0.7870 | 0.7951 |
| 263.9 | Unspecified protein-calorie malnutrition | 0.5696 | 0.6341 | 0.6104 | 0.6548 |
| 272.0 | Pure hypercholesterolemia | 0.5914 | 0.6726 | 0.5811 | 0.6679 |
| 272.4 | Other and unspecified hyperlipidemia | 0.6992 | 0.7330 | 0.7017 | 0.7233 |
| 274.9 | Gout, unspecified | 0.6506 | 0.6937 | 0.6644 | 0.7028 |
| 276.0 | Hyperosmolality and/or hypernatremia | 0.5548 | 0.6521 | 0.6403 | 0.6321 |
| 276.1 | Hyposmolality and/or hyponatremia | 0.5835 | 0.6052 | 0.5985 | 0.5831 |
| 276.2 | Acidosis | 0.5314 | 0.6112 | 0.5921 | 0.5706 |
| 276.5 | Volume depletion disorder | 0.7679 | 0.7510 | 0.6397 | 0.7286 |
| 276.51 | Dehydration | 0.5220 | 0.6071 | 0.6071 | 0.6121 |
| 276.52 | Hypovolemia | 0.5610 | 0.5898 | 0.6464 | 0.6117 |
| 276.7 | Hyperpotassemia | 0.6037 | 0.6646 | 0.6645 | 0.6469 |
| 276.8 | Hypopotassemia | 0.5368 | 0.5989 | 0.5930 | 0.5889 |
| 278.00 | Obesity, unspecified | 0.6322 | 0.6466 | 0.6075 | 0.6763 |
| 280.9 | Iron deficiency anemia, unspecified | 0.5936 | 0.5849 | 0.6430 | 0.6502 |
| 285.1 | Acute posthemorrhagic anemia | 0.5498 | 0.5809 | 0.5794 | 0.6176 |
| 285.21 | Anemia in chronic kidney disease | 0.7542 | 0.8351 | 0.7897 | 0.8087 |
| 285.29 | Anemia of other chronic disease | 0.4657 | 0.6020 | 0.6177 | 0.5890 |
| 285.9 | Anemia, unspecified | 0.5602 | 0.5307 | 0.5603 | 0.5538 |
| 287.5 | Thrombocytopenia, unspecified | 0.5899 | 0.6201 | 0.6124 | 0.6306 |
| 293.0 | Delirium due to conditions classified elsewhere | 0.5294 | 0.6570 | 0.6532 | 0.6454 |
| 300.00 | Anxiety state, unspecified | 0.5928 | 0.6682 | 0.6180 | 0.6318 |
| 305.1 | Tobacco use disorder | 0.6916 | 0.6614 | 0.6158 | 0.6972 |
| 311 | Depressive disorder, not elsewhere classified | 0.5535 | 0.6048 | 0.6120 | 0.6267 |
| 327.23 | Obstructive sleep apnea (adult)(pediatric) | 0.7468 | 0.7254 | 0.6830 | 0.7481 |
| 357.2 | Polyneuropathy in diabetes | 0.7000 | 0.8151 | 0.7857 | 0.8439 |
| 401.9 | Unspecified essential hypertension | 0.6664 | 0.6500 | 0.5712 | 0.5960 |
| 403.90 | Hypertensive chronic kidney disease, unspecified, with chronic kidney disease stage I through stage IV, or unspecified | 0.7586 | 0.7355 | 0.7660 | 0.7324 |
| 403.91 | Hypertensive chronic kidney disease, unspecified, with chronic kidney disease stage V or end stage renal disease | 0.7252 | 0.7905 | 0.7657 | 0.8068 |
| 410.71 | Subendocardial infarction, initial episode of care | 0.6521 | 0.6632 | 0.6360 | 0.6756 |
| 412 | Old myocardial infarction | 0.7021 | 0.7207 | 0.6497 | 0.7080 |
| 414.00 | Coronary atherosclerosis of unspecified type of vessel, native or graft | 0.8427 | 0.8086 | 0.8103 | 0.7859 |
| 414.01 | Coronary atherosclerosis of native coronary artery | 0.6674 | 0.7394 | 0.7180 | 0.7582 |
| 416.8 | Other chronic pulmonary heart diseases | 0.6518 | 0.7186 | 0.7114 | 0.7265 |
| 424.0 | Mitral valve disorders | 0.5332 | 0.6812 | 0.6344 | 0.6655 |
| 424.1 | Aortic valve disorders | 0.6559 | 0.6905 | 0.6332 | 0.6999 |
| 425.4 | Other primary cardiomyopathies | 0.6772 | 0.6951 | 0.6118 | 0.6922 |
| 427.1 | Paroxysmal ventricular tachycardia | 0.5593 | 0.6078 | 0.6272 | 0.6459 |

| | | | | | |
|---|---|---|---|---|---|
| 427.31 | Atrial fibrillation | 0.6286 | 0.6976 | 0.6674 | 0.6946 |
| 427.89 | Other specified cardiac dysrhythmias | 0.5266 | 0.5613 | 0.5654 | 0.5594 |
| 428.0 | Congestive heart failure, unspecified | 0.6646 | 0.7179 | 0.6848 | 0.7066 |
| 428.22 | Chronic systolic heart failure | 0.7801 | 0.7839 | 0.7929 | 0.7954 |
| 428.23 | Acute on chronic systolic heart failure | 0.7763 | 0.7845 | 0.8008 | 0.7989 |
| 428.32 | Chronic diastolic heart failure | 0.7178 | 0.7746 | 0.7159 | 0.7769 |
| 428.33 | Acute on chronic diastolic heart failure | 0.7469 | 0.7813 | 0.7703 | 0.7954 |
| 443.9 | Peripheral vascular disease, unspecified | 0.6569 | 0.7132 | 0.6913 | 0.7265 |
| 458.29 | Other iatrogenic hypotension | 0.5812 | 0.5815 | 0.6068 | 0.6260 |
| 458.9 | Hypotension, unspecified | 0.5155 | 0.5112 | 0.5865 | 0.5688 |
| 486 | Pneumonia, organism unspecified | 0.5949 | 0.6036 | 0.5912 | 0.6069 |
| 491.21 | Obstructive chronic bronchitis with (acute) exacerbation | 0.7516 | 0.7610 | 0.7172 | 0.7696 |
| 493.90 | Asthma, unspecified type, unspecified | 0.6958 | 0.6102 | 0.5686 | 0.6018 |
| 496 | Chronic airway obstruction, not elsewhere classified | 0.6621 | 0.6560 | 0.6130 | 0.6462 |
| 507.0 | Pneumonitis due to inhalation of food or vomitus | 0.5925 | 0.6144 | 0.5990 | 0.6162 |
| 511.9 | Unspecified pleural effusion | 0.5607 | 0.5938 | 0.5534 | 0.5818 |
| 518.0 | Pulmonary collapse | 0.5263 | 0.5404 | 0.5684 | 0.5465 |
| 518.5 | Pulmon insuff fl tra n surg | 0.5544 | 0.5812 | 0.5122 | 0.5895 |
| 518.81 | Acute respiratory failure | 0.5955 | 0.6276 | 0.5857 | 0.5918 |
| 530.81 | Esophageal reflux | 0.5689 | 0.5534 | 0.5535 | 0.5588 |
| 571.2 | Alcoholic cirrhosis of liver | 0.7212 | 0.7590 | 0.6885 | 0.8020 |
| 571.5 | Cirrhosis of liver without mention of alcohol | 0.7004 | 0.7167 | 0.7197 | 0.7624 |
| 578.9 | Hemorrhage of gastrointestinal tract, unspecified | 0.6031 | 0.6018 | 0.5759 | 0.6406 |
| 584.5 | Acute kidney failure with lesion of tubular necrosis | 0.6060 | 0.6340 | 0.5891 | 0.5945 |
| 584.9 | Acute kidney failure, unspecified | 0.6011 | 0.6489 | 0.6340 | 0.6317 |
| 585.6 | End stage renal disease | 0.7728 | 0.8209 | 0.7932 | 0.8125 |
| 585.9 | Chronic kidney disease, unspecified | 0.7504 | 0.6987 | 0.7306 | 0.6872 |
| 599.0 | Urinary tract infection, site not specified | 0.6031 | 0.6118 | 0.6142 | 0.5985 |
| 600.00 | Hypertrophy (benign) of prostate without urinary obstruction and other lower urinary tract symptom (LUTS) | 0.7400 | 0.7021 | 0.7021 | 0.7276 |
| 707.03 | Pressure ulcer, lower back | 0.6176 | 0.6465 | 0.6646 | 0.6350 |
| 733.00 | Osteoporosis, unspecified | 0.5918 | 0.6142 | 0.5954 | 0.6635 |
| 995.91 | Sepsis | 0.5804 | 0.6758 | 0.6604 | 0.6339 |
| 995.92 | Severe sepsis | 0.6323 | 0.6570 | 0.6385 | 0.6146 |
| 997.1 | Cardiac complications, not elsewhere classified | 0.6255 | 0.7123 | 0.6041 | 0.7188 |
| 998.11 | Hemorrhage complicating a procedure | 0.6072 | 0.5940 | 0.5966 | 0.6336 |
| 998.59 | Other postoperative infection | 0.6530 | 0.6786 | 0.5526 | 0.6218 |
| Average | | 0.6369 | 0.6693 | 0.6488 | 0.6718 |
